# Solar Cells Based on PTB7-Fx: PC_71_BM Active Layer Processed with Two Types of Solvent Additives and Sputtered Ag Top-Electrode

**DOI:** 10.3390/ijms27094064

**Published:** 2026-05-01

**Authors:** Georgy Grancharov, Rositsa Gergova, Georgi Popkirov, Hristosko Dikov, Marushka Sendova-Vassileva

**Affiliations:** 1 Institute of Polymers, Bulgarian Academy of Sciences, Acad. G. Bontchev Str. Bl. 103A, 1113 Sofia, Bulgaria; 2 National Centre of Excellence Mechatronics and Clean Technologies, 8 Blvd. Kliment Ohridski, 1756 Sofia, Bulgaria; 3 Central Laboratory of Solar Energy and New Energy Sources, Bulgarian Academy of Sciences, 72 Tzarigradsko Chaussee, 1784 Sofia, Bulgaria; rositsa.gergova@gmail.com (R.G.); popkirov@phys.bas.bg (G.P.); dikov@phys.bas.bg (H.D.); marushka@phys.bas.bg (M.S.-V.); 4 Center of Competence of Mechatronics and Clean Technologies—MIRACle, “Acad. G. Bontchev” Str. 4, 1113 Sofia, Bulgaria

**Keywords:** organic solar cells, bulk heterojunction active layers, organic photovoltaics, current density–voltage characteristics, impedance spectroscopy, sputtered top-electrode, solvent additives

## Abstract

Organic-type solar cells containing an active layer of block copolymer donor PTB7-Fx (x = 0, 20, and 100), based on benzo [1,2-b:4,5-b’]dithiophene and variably fluorinated thieno [3,4-b]thiophene units, and fullerene acceptor [6,6]phenyl-C_71_-methylbutyrate, were constructed. The active layer thin film of the solar cells was obtained from a dichlorobenzene solution at an established concentration via spin-coating of the donor–acceptor mixture in the presence of solvent additives such as 3% diiodooctane and 1% triethyl phosphate. Organic photovoltaic elements with normal device architecture were prepared on glass substrates using an indium tin oxide anode, a spin-coated hole transporting layer of poly(ethylene dioxythiophene):polystyrenesulfonate, the aforementioned active layer, followed by an electron transporting layer of zinc oxide nanoparticles, and finally a magnetron sputtered silver (Ag) top-electrode. The optical properties, thin film morphology, and the thickness of the active layers were investigated. Additionally, current density–voltage characteristics and impedance spectra of photovoltaic devices were measured. It was found that PTB7-Fx:PC_71_BM-based solar cells processed in the presence of two types of solvent additives, diiodooctane and triethyl phosphate, with a sputtered Ag top-electrode display similar absorption and quantum efficiency spectra, as well as comparable current density–voltage characteristics and efficiencies to the same devices fabricated without additives. The diiodooctane solvent additive preferably dissolves the fullerene component and has a positive effect on fill factor enhancement, impedance spectra improvement, and amelioration in charge carrier transport and collection, whereas the triethyl phosphate solvent additive preferentially dissolves the copolymer donor and has a more pronounced impact on the refined morphology of the thin film active layers.

## 1. Introduction

Organic photovoltaics (PVs) based on an active layer of conjugated polymer donors and organic fullerene acceptors have drawn the attention of the scientific community in the recent decade. Developed as an alternative to inorganic silicon-based solar cells, organic solar cells offer easy deposition on flexible substrates, low-cost fabrication, and possibilities for chemical engineering [1,2,3]. A plethora of conjugated (co)polymers has been developed for the donor part of the bulk heterojunction (BHJ) active layer, containing single building moieties such as para-phenylenevinylene and 3-hexylthiophene, or alternatively using a combination of two moieties for finely tuned band gaps, such as fluorene, carbazol, cyclopentadithiophene, benzothiadiazole, benzodithiophene, and thienothiophene [4,5,6]. Different fullerene C_60_ and C_70_ derivatives have been utilized as the main building entities for the acceptor part of the BHJ active layer. Until recently, rylenediimide polymers and non-fullerene molecules have been developed, examined, and applied [7,8,9]. Undoubtedly, the organic PVs with an active layer combining the donor polymer poly(3-hexylthiphene) and the fullerene acceptor [6,6]phenyl-C_61_-methylbutyrate (PC_61_BM) is the most explored and investigated system nowadays [10,11]. Recent efforts to optimize the efficiency of organic PVs have brought to our attention the progress of ternary solar cells containing a second donor or acceptor material based on a single bulk heterojunction structure [12,13,14], or additionally, tandem solar cells where two or more sub-cells are stacked and connected in series or parallel in one multi-junction cell configuration [15].

A conjugated polymer showing good efficiency and possessing a low band gap is PTB7–Fx (x = 0 ÷ 100), (poly[[4,8-bis[(2-ethylhexyl)oxy]benzo[1,2-b:4,5-b′]dithiophene-2,6-diyl]-alt-[2-[(2-ethylhexyl)-carbonyl]-thieno[3,4-b]thiophenediyl]]-alt-[2-[(2-ethylhexyl)-3-fluoro-carbonyl-thieno[3,4-b]thiophenediyl]]). That donor copolymer was usually mixed with a strong acceptor compound such as methyl ester C_71_ phenyl butyrate (PC_71_BM) for the preparation of the BHJ active layer producing organic PVs with a power conversion efficiency (PCE) up to 6.0–8.2% for devices with normal architecture [16,17], and respectively, up to 8.0–9.2% PCE in the cases of devices with an inverted architecture [18]. It was found that the molecular weight and the degree of fluorination of the thienothiophene unit of the PTB7-Fx copolymer appear to be highly necessary for good device performance. They especially determine the important factors for the solar cells’ parameters as a lower fullerene domain size, higher short circuit current, and lower HOMO level of the donor copolymer [19,20,21].

Additionally, the selection of the proper host solvent and the type of BHJ solvent additive causes essential influence on the behavior of the prepared PV elements as well. The boiling point of host solvent leads to drastic changes in the BHJ morphology because of the solubility difference in the PTB7-Fx copolymer donor and the fullerene acceptor, and correspondingly in solar cell performance. Thus, the highest device performance is exhibited by such material combinations where the donor and acceptor components are of similar and sufficiently high solubility in the chosen solvent [22,23]. 1,8-Diiodooctane (DIO) as a widely used processing additive facilitates specific interactions in the BHJ film by increasing the interfacial area and exciton dissociation, optimizes intermixed film morphology via smaller length scales and improves the crystalline formation leading to increased charge transport. It preferably dissolves the fullerene component and in this way reduces the size of the donor–acceptor domains and enlarges the donor–acceptor interface [24,25,26]. Another solvent additive frequently applied in the practice is chloronaphthalene (CN). It changes the film morphology of the BHJ layer via reducing the phase separation between the donor and acceptor wherever the crystallization or the molecular order of the polymer donor is not affected but the size of the large fullerene aggregates is reduced. The outcome of CN additive influence is an increased ratio of the donor polymer to the fullerene derivative and high hole mobility throughout the donor polymer in the BHJ layer [27]. Diphenyl ether (DPE) is also employed as a processing additive and promotes the formation of nano-fibrillar networks and ordered packing of the PTB7:PC_71_BM active layer, facilitates charge transport at long distances, and suppresses charge recombination [28].

In this contribution we investigated and determined the important parameters of photovoltaic elements based on the PTB7-Fx: PC_71_BM active layer processed in the absence and the presence of two types of solvent additives such as the commonly used 1,8-diiodooctane and triethyl phosphate (TEP), which is still not applied in the practice additive, aiming for efficient power conversion of solar energy in organic PVs. TEP was chosen as an additive solvent due to its high boiling point and good solubility towards most of the organic compounds and polymers. TEP has been already used as an efficient co-solvent in electrolytes for lithium-ion batteries, as a co-solvent in electrolytes for the electrochemical performance of supercapacitors, and as an antisolvent for organometallic halide perovskite solar cells [29,30,31]. The thin active layer films were prepared in a donor:acceptor ratio of 1:1.5 in the dichlorobenzene solution with the addition of 1% TEP or 3% of DIO solvent additives via spin-coating. The main goal was the investigation of the behavior of the two types of solvent additives, TEP or DIO, which were able to dissolve preferably either the copolymer donor or the fullerene component, respectively. Test devices using normal architecture were constructed using an ITO anode, a poly(ethylene dioxythiophene): polystyrenesulfonate (PEDOT: PSS) hole transporting layer, a PTB7-Fx: PC_71_BM: solvent additive active layer, a ZnO nanoparticles electron transporting layer, and a magnetron sputtered Ag top-electrode. The optical absorption spectra were used for characterization of the modified active layers, the control of BHJ film morphology achieved by two solvent additives were analyzed via atomic force microscopy (AFM). Current density–voltage (J-V) characteristics of the obtained organic solar cells were measured and their PCEs were evaluated. Impedance spectra of the fabricated PVs were obtained and their equivalent circuits were fitted. To the best of our knowledge, TEP was not used as a solvent additive in organic PVs and, herewith, its behavior on the properties of solar cells was investigated and compared to solar cells prepared without and containing a DIO solvent additive.

## 2. Results and Discussion

**Figure 1:**
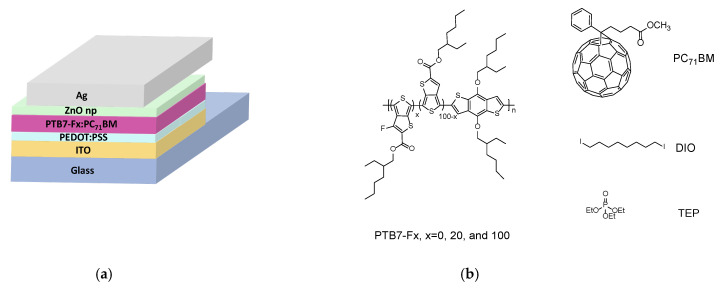
Structure of (**a**) prepared photovoltaic devices and (**b**) active layer ingredients.

Three donor-type block copolymers PTB7–Fx (x = 0, 20, 100) based on benzo[1,2-b:4,5-b’]dithiophene and variably fluorinated thieno[3,4-b]thiophene units were synthesized and characterized. They were mixed with the acceptor compound PC_71_BM in the absence or the presence of two types of solvent additives such as 3% DIO and 1% TEP, respectively, in the dichlorobenzene solvent for the preparation of the organic photoelements active layer. It was sandwiched between a hole transporting layer of PEDOT:PSS and an electron transporting layer of ZnO nanoparticles for the fabrication of PVs with a magnetron sputtered Ag top-electrode possessing normal architecture (Figure 1). The thickness of the active layer of PVs with different PTB7-Fx copolymers, fullerene acceptor PC_71_BM, and two types of solvent additives showed consistent thickness in the range from 75 ± 6 nm to 87 ± 8 nm as revealed by profilometer measurements. For statistical purposes, about 16 photovoltaic devices with identical active layers were fabricated from PTB7-Fx: PC_71_BM-based solar cells processed in the absence or in the presence of two types of solvent additives, DIO and TEP.

**Figure 2:**
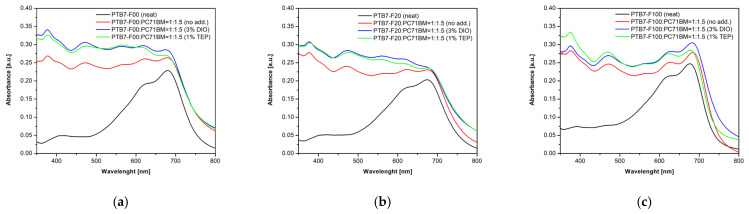
Absorption spectra of neat copolymer, of BHJ active layer in the presence of acceptor compound PC_71_BM, and the corresponding mixtures of active layer with solvent additives such as 3% DIO and 1% TEP, respectively: (**a**) x = 0, PTB7-F00; (**b**) x = 20, PTB7-F20; and (**c**) x = 100, PTB7-F100.

The absorption spectra of the thin films of neat copolymers PTB7-Fx, of their mixtures with the acceptor compound PC_71_BM for obtaining BHJ active layers, and the corresponding mixtures of active layers in the presence of two types of solvent additives such as 3% DIO and 1% TEP, respectively, are shown in Figure 2a–c. The three donor block copolymers and their BHJ mixtures with fullerene derivatives exhibited identical absorption spectra in the range 350–800 nm. The minimal amount of solvent additives DIO and TEP added to the active layer caused negligible increases in their absorption spectra in the measured range compared to the layer without additives. A decrease in the intensity of the vibronic shoulder in the range 575–725 nm can be detected for all active films containing the TEP additive. This can be explained by the strong dissolution properties of the TEP additive towards the donor polymer phase and its decreased possibility for further structural organization.

**Figure 3:**
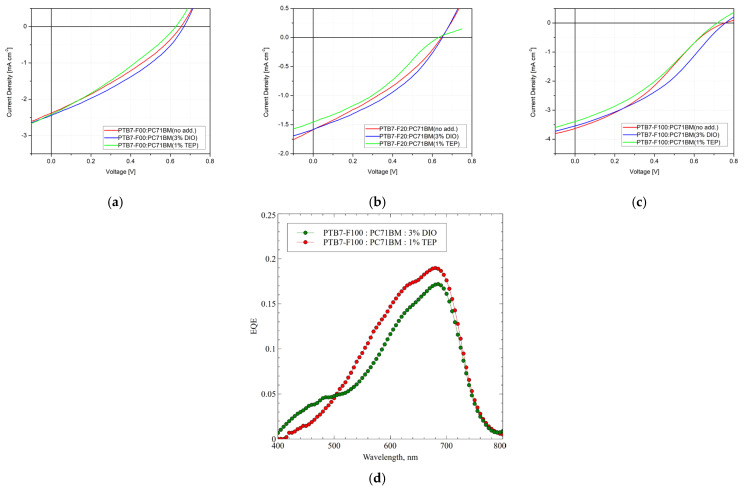
Current density–voltage characteristics of organic solar cells prepared from active layer of PTB7-Fx donor copolymer and PC_71_BM acceptor, in the absence or the presence of solvent additives such as 3% DIO and 1% TEP, respectively: (**a**) x = 0, PTB7-F00; (**b**) x = 20, PTB7-F20; and (**c**) x = 100, PTB7-F100. (**d**) EQE spectra of two PTB7-F100: PC_71_BM solar cells prepared in the presence of DIO and TEP solvent additives.

In Figure 3 are presented the current density–voltage characteristics of the prepared organic solar cells with normal structures of ITO/PEDOT:PSS/active layer/ZnO np/Ag and active layer of PTB7-Fx: PC71BM constructed in the absence or the presence of solvent additives such as 3% DIO and 1% TEP, respectively.

The detailed characterizations of the photovoltaic parameters of the investigated photovoltaic cells prepared from the active layer of the PTB7-Fx donor copolymer, PC_71_BM acceptor, and the absence or the presence of solvent additives such as 3% DIO and 1% TEP, respectively, are presented in Table 1.

The current density–voltage characteristics of organic solar cells fabricated from the conjugated donor copolymer PTB7-F00 without fluorine content in the thieno[3,4-b]thiopehene unit and mixed with the PC_71_BM acceptor compound are presented in Figure 3a. The BHJ active layer prepared without solvent additives is presented by the red J-V curve and showed photovoltaic parameters as follows: open circuit voltage (Voc)—0.655 V, short circuit current density (Jsc)—2.44 mA/cm^2^, and fill factor (FF)—31.7% which represents a PCE of value 0.51%. In the case of photovoltaic cells with the BHJ active layer prepared in the presence of 3% DIO solvent additive (blue J-V curve), the photovoltaic characteristics revealed Voc—0.669 V, Jsc—2.38 mA/cm^2^, and FF—36.9%, which represents a PCE of value 0.59%. Photovoltaic elements with the BHJ active layer fabricated in the presence of the 1% TEP solvent additive (green J-V curve) possessed Voc—0.629 V, Jsc—2.41 mA/cm^2^, and FF—26.9%, which represents a PCE of value 0.41%. A partial increase in the electrical parameters of organic cells prepared with the DIO solvent additive was found due to the increased FF and Voc, and correspondingly, a slight decrease in the photovoltaic parameters of organic PVs fabricated with the TEP solvent additive due to decreases in FF and Voc.

For the solar cells constructed of the donor copolymer PTB7-F20 with 20% fluorine content and mixed with the PC_71_BM acceptor compound, the values of photovoltaic parameters are shown in Figure 3b and they reveal for the BHJ active layer constructed without solvent additives (red J-V curve) an open circuit voltage of 0.651 V, short circuit current density—1.59 mA/cm^2^, and fill factor—36.1%, yielding a PCE of 0.37%. The expected melioration in electric parameters was not observed even by the partial increase in the fluorine content with 20% in the donor copolymer which can be explained by the simultaneous decrease in its molecular weight [20,21]. Analogous was the trend in the case of photovoltaic cells with the BHJ active layer prepared in the presence of 3% DIO (blue J-V curve) where the photovoltaic characteristics revealed that Voc—0.651 V, Jsc—1.58 mA/cm^2^, and FF—41.0%, which represents a PCE of value 0.42%; also, in the case of photoelements with the BHJ active layer fabricated in the presence of 1% TEP (green J-V curve) possessed Voc—0.631 V, Jsc—1.45 mA/cm^2^, and FF—31.4%, which represents a PCE of value 0.29%. It was noticed that the behavior of both solvent additives over the electrical characteristics was the same as in the previous case for PTB7-F00-based photoelements.

Similarly, the electrical parameters of organic solar cells with 100% fluorine content and also mixed with PC_71_BM acceptor compound were investigated (Figure 3c). An improvement in the photovoltaic parameters was observed showing open circuit voltage of 0.757 V, short circuit current density—3.63 mA/cm^2^, and fill factor—34.4%, yielding a PCE of 0.95%. The increase in electrical characteristics was due to the influence of both occasions as an attachment of the maximal amount of fluorine units to thieno[3,4-b]thiopehene and a higher molecular weight of the PTB7-F100 copolymer leading to the optimal structural morphology of the BHJ active layer [20,21]. In the case of photovoltaic cells with the BHJ active layer prepared in the presence of 3% DIO (blue J-V curve) the photovoltaic characteristics revealed Voc—0.757 V, Jsc—3.54 mA/cm^2^, and FF—38.6%, which represents a PCE of value 1.03%. Photovoltaic elements with the BHJ active layer fabricated in the presence of the 1% TEP solvent additive (green J-V curve) possessed opening Voc—0.715 V, Jsc—3.39 mA/cm^2^, and FF—30.5%, which represents a PCE of value 0.74%. The tendency of an increase in the electrical characteristics of the organic cells prepared with the DIO solvent additive, and correspondingly a decrease in the photovoltaic characteristics of organic PVs fabricated with the TEP solvent additive herein, was also preserved. It was obvious that the DIO solvent additive which preferably dissolves the fullerene component caused a slight increase in the FF and Voc of organic PVs, whereas the TEP solvent additive which preferentially dissolves the copolymer donor caused slight decreases in the FF and Voc of organic PVs.

Figure 3d shows the spectra of the external quantum efficiency (EQE) of the cells with 100% fluorine content and the two different additives. It can be seen that the intensity and the shape of the spectra is similar for the two cells and correlates with the optical absorption spectra shown in Figure 2c. In the case of the DIO addition there is an increase in the spectral sensitivity in the blue part of the spectrum and a decrease in the red spectral region.

As obtained PTB7-Fx: PC_71_BM-based solar cells prepared in the presence of two types of solvent additives possess sputtered Ag top-electrodes. It was noticed that they displayed comparable current–voltage characteristics and efficiencies to devices with standard evaporated Al top-electrodes [32] due to the application of an electron transporting layer of zinc oxide nanoparticles. It prevents and preserves the entity of the device’s bulk heterojunction active layer from damages during the sputtering process and improves the S-shape kink of J-V curves when it was incorporated as ETL in the photovoltaic devices [33,34].

**Figure 4:**
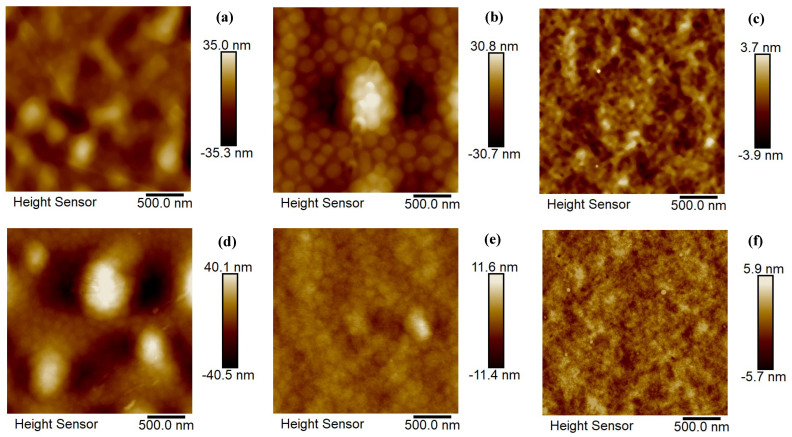
PFT-AFM height images of the active films for organic solar cells prepared from mixture of: (**a**) PTB7-F00: PC_71_BM: 3% DIO, (**b**) PTB7-F20: PC_71_BM: 3% DIO, (**c**) PTB7-F100: PC_71_BM: 3% DIO, (**d**) PTB7-F00: PC_71_BM: 1% TEP, (**e**) PTB7-F20: PC_71_BM: 1% TEP, and (**f**) PTB7-F100: PC_71_BM: 1% TEP.

Further, the structural morphology of the BHJ layer based on PTB7-Fx copolymers and the PC_71_BM acceptor compound was analyzed via AFM characterizations of the BHJ thin layer of organic solar cells containing two types of solvent additives such as 1% TEP and 3% DIO (Figure 4).

Isolated domains of size 100–400 nm and the absence of a continuous interpenetrating network are detected in the AFM height image for the BHJ active layer of PTB7-F00: PC_71_BM prepared in the presence of the DIO solvent additive shown on Figure 4a. Similar morphology but smaller isolated domains of size 100–250 nm are typical for the active layer of PTB7-F20: PC_71_BM fabricated from DIO participation (Figure 4b). In the image of the BHJ active layer of PTB7-F100: PC_71_BM (Figure 4c) processed using the DIO solvent additive were observed smaller domains of size 50–150 nm and the presence of a continuous interpenetrating network. Additionally, the signatures of nanofibrillar domains were also noticed for the PTB7-F100: PC_71_BM film morphology. Such a structural morphology type is favorable for efficient charge transport, improvement of current–voltage characteristics, and, consequently, better PCE of photoelements. Analogously, the isolated domains of narrow polydispersity and size 50–150 nm were discovered in the AFM height image for the BHJ active layer of PTB7-F00: PC_71_BM prepared in the presence of the TEP solvent additive shown in Figure 4d. For the morphology of the active layer of PTB7-F20: PC_71_BM fabricated from TEP participation (Figure 4e) were found similar isolated domains of size 50–200 nm and the absence of a continuous interpenetrating network. In the image of the BHJ active layer of PTB7-F100: PC_71_BM (Figure 4f) processed using the TEP solvent additive were observed even smaller domains of size 30–100 nm and the presence of a continuous interpenetrating network. The signatures of nanofibrillar domains were also noticed for the later film morphology which is useful for efficient charge transport, improvement of electrical characteristics and higher PCE. In order to compare the BHJ active layer morphology of both PTB7-Fx: PC_71_BM mixtures in the presence of DIO and TEP solvent additives, it can be outlined that both active layers possess similar structural BHJ morphology. They both possess domains of optimal sizes at about 15–50 nm which are close to the sizes of exciton diffusion length, and are prerequisites for the efficient performance of PV devices, and opposite, an increase in the domain sizes leads to a negative impact on the work of PVs. That is why the as obtained size of nanofibrillar domains benefits efficient charge carrier transport and collection, enhanced current–voltage characteristics and power conversion efficiency. The structural morphology of BHJ with the participation of the TEP solvent additive seems more favorable, showing a continuous interpenetrating network and smaller domains with even lower values for electrical parameters.

**Figure 5:**
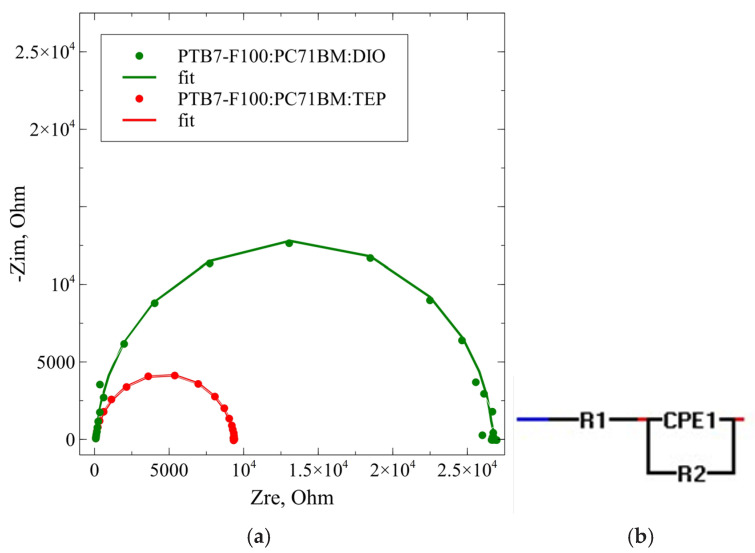
(**a**) Impedance spectra of organic solar cells prepared from BHJ active layer of PTB7-F100:PC_71_BM in the presence of DIO and TEP, respectively, and (**b**) equivalent circuit used for the fitting of the spectra.

Impedance spectra (IS) as a tool for measuring the efficient work of solar cells were also investigated and they usually were obtained under one sun illumination at short circuit conditions (Figure 5).

Figure 5a shows the Nyquist plot of the IS measurement for typical organic solar cells with the addition of DIO or TEP to the active layer solution of PTB7-F100:PC_71_BM. The equivalent circuit used for the fitting of the spectra is shown as well (Figure 5b). It is a simple CPE (constant phase element) circuit with a series and bulk resistance. The CPE stands in place of the capacitance and represents a distributed capacitance connected to the inhomogeneity of the interface. The series resistance (R1) is comparatively small for both cells with a value of 4.8 Ohm.cm^2^ for the element with DIO and 2.7 Ohm.cm^2^ for the one with TEP. The bulk or recombination resistance (R2) is almost three times greater for the cell with DIO as can be seen from the diameter of the semicircles in the figure. This gives an advantage to the cell with the DIO-modified active layer as there is a much smaller carrier recombination before extraction in it. In both cases the n parameter of the CPE element is close to one, meaning that the semicircles are not much depressed, which is evidence that the inhomogeneity of the interface is not great. The values of the fit parameters for the elements of the equivalent circuit are given in Table 2.

## 3. Materials and Methods

### 3.1. Materials

Glass/indium tin oxide (ITO)-patterned substrates were supplied by Ossila, UK. All the solvents as acetone (99.8%), isopropanol (99.8%), and methanol (99.9%) were purchased from Fisher Chemical, UK, and used as received. Donor block copolymers PTB7–Fx (x = 0, 20, 100), (poly[[4,8-bis[(2-ethylhexyl)oxy]benzo[1,2-b:4,5-b′]dithiophene-2,6-diyl]-alt-[2-[(2-ethylhexyl)-carbonyl]-thieno[3,4-b]thiophenediyl]]-alt-[2-[(2-ethylhexyl)-3-fluoro-carbonyl-thieno[3,4-b]thiophenediyl]]) were synthesized as described elsewhere [32]. Acceptor material PC_71_BM was purchased from Sollene BV, Groningen, The Netherlands. The interlayer materials as PEDOT:PSS (PVP AI 4083) and ZnO nanoparticles ink suspension (particles size 10–15 nm) were obtained from HC Clevious, Leverkusen, Germany, and SigmaAldrich–Merck, Milwaukee, WI, USA, respectively. Additive solvents as 1,8-diiodooctane (98%) and triethyl phosphate (99%) were acquired from Sigma Aldrich–Merck, Darmstadt, Germany, and used as received.

### 3.2. Instruments and Methods

UV−Vis absorption spectra of active films were measured on a DU 800 (Beckman Coulter, Brea, CA, USA) spectrometer from 300 to 800 nm. AFM height images were obtained on Bruker Dimension Icon microscope (Bruker Corporation, Karlsruhe, Germany) in peak force tapping (PFT) mode exploring silicon nitride cantilevers with a spring constant of ~0.4 N m^−1^ in air. The J–V curves of obtained solar cells were measured with a Keithley 2400 source meter (Keithley, Solon, OH, USA) under halogen lamp illumination. The thickness of the films was obtained by a Talystep profilometer (Taylor Hobson, Aurora, CO, USA). The impedance spectra were measured under short circuit conditions with a Gamry Interface 5000E potentiostat (Gamry Instuments, Warminster, PA, USA) in the frequency range from 1 Hz to 1 MHz under halogen lamp illumination equivalent to one sun.

### 3.3. Devices Fabrication and Characterization

Organic solar cells with normal device architecture were constructed onto glass substrates patterned with transparent anodes of ITO layers. The ITO substrates were cleaned subsequently by ultrasonication in detergent solution, deionized water, acetone, isopropanol, and methanol. Then, substrates were dried by argon flow and treated in UV ozone chamber. Thin hole transporting layer of PEDOT:PSS was spin-coated and thermally annealed at 120 °C for 30 min in air. Next, organic active layer prepared at 1:1.5 mass ratio of the donor: acceptor mixture in dichlorobenzene solution at 25 mg mL^−1^ and the addition of 3% DIO or 1% TEP, respectively, was deposited by spin-coating in glove-box. The electron transporting layer of ZnO nanoparticles dispersed in isopropanol were applied over the active layer via spin-coating of 40 nm thickness. Finally, silver cathode layer of 200 nm thickness was deposited by magnetron sputtering. A 3″ Ag target was used for the magnetron sputtering; the layers were obtained using a power of 200 W and argon pressure of 0.5 Pa for 5 min duration of the deposition. The current density–voltage characteristics of organic PV elements were measured under a halogen lamp providing illumination 100 mW cm^−2^ light intensity and a Si photodiode applied as a reference. The active area of the photovoltaic devices was 0.04 cm^2^ and shadow mask was not used during the measurement of the current density–voltage and spectral characteristics.

## 4. Conclusions

Donor copolymers PTB7-Fx, where (x = 0, 20 and 100%) were blended with the fullerene derivative PC_71_BM in the presence of two types of solvent additives such as 3% DIO or 1% TEP in dichlorobenzene solution at established concentrations, aimed to prepare an active layer for BHJ organic solar cells. Organic photovoltaic elements with normal device architecture were prepared onto glass substrates using an ITO anode, a spin-coated hole transporting layer of PEDOT:PSS, the above mentioned active layer, followed by an electron transporting layer of zinc oxide nanoparticles, and finally a magnetron sputtered Ag top-electrode. It was found that the as obtained BHJ active layers of PTB7-Fx-based solar cells processed with two types of solvent additives, 3% diiodooctane or 1% triethyl phosphate, with a sputtered Ag top-electrode display similar absorption and quantum efficiency spectra, and comparable current density–voltage characteristics and efficiencies to the same devices but fabricated without additives. The diiodooctane solvent additive preferably dissolves the fullerene component and has a positive effect over fill factor amendment, impedance spectra improvement, amelioration in charge carrier transport and collection, whereas the triethyl phosphate solvent additive preferentially dissolves the copolymer donor and has a more pronounced impact on the refined morphology of thin film active layers. The application of an electron transporting layer of zinc oxide nanoparticles prevents and preserves the entity of the device’s bulk heterojunction active layer from damages during the sputtering process.

## Figures and Tables

**Table 1:** Photovoltaic parameters of solar cells.

Type of Active Layer	Voc, V	Jsc, mA/cm^2^	FF, %	PCE, %
PTB7-F00: PC_71_BM	0.655	2.44	31.7	0.51
PTB7-F00: PC_71_BM: 3% DIO	0.669	2.38	36.9	0.59
PTB7-F00: PC_71_BM: 1% TEP	0.629	2.41	26.9	0.41
PTB7-F20: PC_71_BM	0.651	1.59	36.1	0.37
PTB7-F20: PC_71_BM: 3% DIO	0.651	1.58	41.0	0.42
PTB7-F20: PC_71_BM: 1% TEP	0.631	1.45	31.4	0.29
PTB7-F100: PC_71_BM	0.757	3.63	34.4	0.95
PTB7-F100: PC_71_BM: 3% DIO	0.757	3.54	38.6	1.03
PTB7-F100: PC_71_BM: 1% TEP	0.715	3.39	30.5	0.74

**Table 2:** Fit parameters of the impedance spectra of PTB7-F100:PC_71_BM solar cells in the presence of DIO and TEP solvent additives.

Type of Active Layer	R1, Ω	R2, Ω	CPE1, nF	n1
PTB7-F100: PC_71_BM: 3% DIO	121	26601	4.92	0.974
PTB7-F100: PC_71_BM: 1% TEP	67	9294	7.16	0.934

## Data Availability

The original contributions presented in this study are included in the article. Further inquiries can be directed to the corresponding author.

## References

[1] Guo X., Baumgarten M., Müllen K. (2013). Designing pi-conjugated polymers for organic electronics. Prog. Polym. Sci..

[2] Etxebarria I., Ajuria J., Pacios R. (2015). Solution-processable polymeric solar cells: A review on materials, strategies and cell architectures to overcome 10%. Org. Electron..

[3] Leo K. (2017). Elementary processes in organic photovoltaics. Advances in Polymer Science.

[4] Kularatne R.S., Magurudeniya H.D., Sista P., Biewer M.C., Stefan M.C. (2013). Donor-acceptor semiconducting polymers for organic solar cells. J. Polym. Sci. Part A Polym. Chem..

[5] Peng Q., Liang T., Feng K., Méndez-Vilas A. (2013). Design of low bandgap conjugated polymers for organic solar cell. Materials and Processes for Energy: Communicating Current Research and Technological Developments.

[6] Amna B., Siddiqi H.M., Hassana A., Ozturk T. (2020). Recent developments in the synthesis of regioregular thiophene-based conjugated polymers for electronic and optoelectronic applications using nickel and palladium-based catalytic systems. RSC Adv..

[7] Ryno S.M., Ravva M.K., Chen X., Li H., Brédas J.-L. (2017). Molecular understanding of fullerene-electron donor interactions in Organic Solar Cells. Adv. Energy Mater..

[8] Cheng P., Zhan X., Yang Y. (2018). Next-generation organic photovoltaics based on non-fullerene acceptors. Nat. Photonics.

[9] Naveed H.B., Ma W. (2018). Miscibility-Driven Optimization of nanostructures in ternary organic solar cells using non-fullerene acceptors. Joule.

[10] Brabec C.H., Gowrisanker S., Halls J.J.M., Laird D., Jia S., Williams S.P. (2010). Polymer-fullerene bulk-heterojunction solar cells. Adv. Mater..

[11] Dutta P., Xie Y., Kumar M., Rathi M., Ahrenkiel P., Galipeau D., Qiao Q., Bommisetty V. (2011). Connecting physical properties of spin-casting solvents with morphology, nanoscale charge transport, and device performance of poly(3-hexylthiophene):phenyl-C_61_-butyric acid methyl ester bulk heterojunction solar cells. J. Photonics Energy.

[12] Ameri T., Khoram P., Min J., Brabec C.J. (2013). Organic ternary solar cells: A review. Adv. Mater..

[13] An Q., Zhang F., Zhang J., Tang W., Deng Z., Hu B. (2016). Versatile ternary organic solar cells: A critical review. Energy Environ. Sci..

[14] Farinhas J., Oliveira R., Ferreira Q., Morgado J., Charas A. (2017). Enhanced efficiency of PTB7: PC_61_ BM organic solar cells by adding a low efficient polymer donor. Int. J. Photoenergy.

[15] Ameri T., Li N., Brabec C.J. (2013). Highly efficient organic tandem solar cells: A follow up review. Energy Environ. Sci..

[16] Liang Y., Xu Z., Xia J., Tsai S.-T., Wu Y., Li G., Ray C., Yu L. (2010). For the bright future- bulk heterojunction polymer solar cells with power conversion efficiency of 7.4%. Adv. Mater..

[17] Chen H.-Y., Hou J., Zhang S., Liang Y., Yang G., Yang Y., Yu L., Wu Y., Li G. (2009). Polymer solar cells with enhanced open-circuit voltage and efficiency. Nat. Photonics.

[18] He Z., Zhong C., Su S., Xu M., Wu H., Cao Y. (2012). Enhanced power-conversion efficiency in polymer solar cells using an inverted device structure. Nat. Photonics.

[19] Wang H., Yu X., Yi C., Ren H., Liu C., Yang Y., Xiao S., Zheng J., Karim A., Cheng S. (2013). Fine-tuning of fluorinated thieno[3,4-b]thiophene copolymer for efficient polymer solar cells. J. Phys. Chem. C.

[20] Guo S., Ning J., Körstgens V., Yao Y., Herzig E., Roth S., Müller-Buschbaum P. (2014). The effect of fluorination in manipulating the nanomorphology in PTB7:PC_71_BM bulk heterojunction systems. Adv. Energy Mater..

[21] He X., Mukherjee S., Watkins S., Chen M., Qin T., Thomsen L., Ade H., McNeill C. (2014). Influence of fluorination and molecular weight on the morphology and performance of PTB7:PC_71_BM solar cells. J. Phys. Chem. C.

[22] Manley E., Strzalka J., Fauvell T., Jackson N., Leonardi M., Eastham N., Marks T., Chen L. (2017). In situ GIWAXS analysis of solvent and additive effects on PTB7 thin film microstructure evolution during spin coating. Adv. Mater..

[23] Guo S., Wang W., Herzig E., Naumann A., Tainter G., Perlich J., Müller-Buschbaum P. (2017). Solvent-morphology-property relationship of PTB7:PC_71_BM polymer solar cells. ACS Appl. Mater. Interfaces.

[24] Wang L., Zhao S., Xu Z., Zhao J., Huang D., Zhao L. (2016). Integrated effects of two additives on the enhanced performance of PTB7:PC_71_BM polymer solar cells. Materials.

[25] Wang J., Liang Z. (2016). Synergetic solvent engineering of film nanomorphology to enhance planar perylene diimide based organic photovoltaics. ACS Appl. Mater. Interfaces.

[26] Lou S.J., Szarko J.M., Xu T., Yu L., Marks T.J., Chen L.X. (2011). Effects of additives on the morphology of solution phase aggregates formed by active layer components of high-efficiency organic solar cells. J. Am. Chem. Soc..

[27] Yi C., Hu X., Liu H.C., Hu R., Hsu C.-H., Zheng J., Gong X. (2015). Efficient polymer solar cells fabricated from solvent processing additive solution. J. Mater. Chem. C.

[28] Zheng Y., Goh T., Fan P., Shi W., Yu J., Taylor A. (2016). Towards efficient thick active PTB7 photovoltaic layers using diphenyl ether as a solvent additive. ACS Appl. Mater. Interfaces.

[29] Wei M., Liang C., Li R., He Y., Rong L., Song X., Li J. (2020). Flame retardant triethyl phosphate/propylene carbonate electrolyte for supercapacitor applications. Int. J. Electrochem. Sci..

[30] Jiang L., Liang C., Li H., Wang Q., Sun J. (2020). Safer triethyl phosphate based electrolyte enables nonflammable and high temperature endurance for lithium ion battery. ACS Appl. Energy Mater..

[31] Zhang P., Gu N., Chen X., Song L., Du P., Chen W.-H., Xiong J. (2021). Triethyl phosphate in an antisolvent: A novel approach to fabricate high-efficiency and stable perovskite solar cells under ambient air conditions. Mater. Chem. Front..

[32] Grancharov G., Gancheva V., Petrov P., Gergova R., Popkirov G., Sendova-Vassileva M. (2018). Optical, film surface and photovoltaic properties of PTB7-Fx- based polymer-organic solar cells. Chem. Pap..

[33] Grancharov G., Atanasova M.-D., Kalinova R., Gergova R., Popkirov G., Dikov C., Sendova-Vassileva M. (2021). Flexible polymer–organic solar cells based on P3HT: PCBM bulk heterojunction active layer constructed under environmental conditions. Molecules.

[34] Gergova R., Sendova-Vassileva M., Popkirov G., Dikov H., Atanasova M.-D., Grancharov G. (2021). Influence of ZnO nanoparticles as electron transport material on the performance and stability of organic solar cells with sputtered Ag back electrodes. J. Phys. Conf. Ser..

